# Effective Feature Selection for Classification of Promoter Sequences

**DOI:** 10.1371/journal.pone.0167165

**Published:** 2016-12-15

**Authors:** Kouser K., Lavanya P. G., Lalitha Rangarajan, Acharya Kshitish K.

**Affiliations:** 1 DoS in Computer Science, Mysore, India; 2 Institute of Bioinformatics and Applied Biotechnology (IBAB), Biotech Park, Electronic City, Bengaluru (Bangalore), Karnataka state, India; 3 Shodhaka Life Sciences Pvt. Ltd., IBAB, Biotech Park, Bengaluru (Bangalore), Karnataka state, India; Harbin Institute of Technology Shenzhen Graduate School, CHINA

## Abstract

Exploring novel computational methods in making sense of biological data has not only been a necessity, but also productive. A part of this trend is the search for more efficient in silico methods/tools for analysis of promoters, which are parts of DNA sequences that are involved in regulation of expression of genes into other functional molecules. Promoter regions vary greatly in their function based on the sequence of nucleotides and the arrangement of protein-binding short-regions called motifs. In fact, the regulatory nature of the promoters seems to be largely driven by the selective presence and/or the arrangement of these motifs. Here, we explore computational classification of promoter sequences based on the pattern of motif distributions, as such classification can pave a new way of functional analysis of promoters and to discover the functionally crucial motifs. We make use of Position Specific Motif Matrix (PSMM) features for exploring the possibility of accurately classifying promoter sequences using some of the popular classification techniques. The classification results on the complete feature set are low, perhaps due to the huge number of features. We propose two ways of reducing features. Our test results show improvement in the classification output after the reduction of features. The results also show that decision trees outperform SVM (Support Vector Machine), KNN (K Nearest Neighbor) and ensemble classifier LibD3C, particularly with reduced features. The proposed feature selection methods outperform some of the popular feature transformation methods such as PCA and SVD. Also, the methods proposed are as accurate as MRMR (feature selection method) but much faster than MRMR. Such methods could be useful to categorize new promoters and explore regulatory mechanisms of gene expressions in complex eukaryotic species.

## Introduction

It is challenging to make sense out of the exponentially increasing biological data, particularly the nucleotide sequences. Efficient, robust, scalable analysis of biological data is the need of the hour as biological data is noisy and high dimension in nature [1]. Many new methods/techniques can now help in the process of extracting meaningful information from the sequences for better understanding of biomedical mechanisms [2] and to attempt solve specific biological problems. Promoter sequences consist of mainly non-coding sequences and usually have multiple transcription factor binding sites (TFBS)/motifs, which consist of specific types of patterns with 5–20 nucleotides [3]. Many researchers have earlier tried to use such features of promoters to predict and/or analyze them [4,5]. We have earlier attempted to analyze promoters using motif-frequency and alignments [6,7]. In this work, we have devised novel computational methods to analyze promoter sequences.

Exploring what constitutes a functional signal or property at the sequence level is the objective of many sequence analysis exercises. Often, classification of segments of sequences is useful for this type of analysis and thus classification techniques have become an integral part of biological data analysis [8]. The biological data is often huge in terms of dimension with comparatively less number of samples posing an inevitable challenge for classification methods to successfully identify classes. Several approaches like Decision Trees (DT), k-Nearest Neighbor (KNN), Support Vector Machine (SVM), Artificial Neural Networks (ANN) have been found effective in the problem of classification of biological data [1]. General nucleotide feature extractions may also not help in comparing promoter sequences from complex eukaryotes. For example, repDNA [9] and repRNA [10] are useful tools for generating multiple features reflecting the physicochemical properties and sequence-order effects of nucleic acids. But, they have been neither designed to use information on TFBSs nor to compare two sets of sequences. Pse-in-One is a useful feature extraction software tool [11]. Pse DAC—General, a component of Pse-in-One, is a tool for finding various feature vectors out of a given DNA sequence. This tool takes as input, a DNA sequence and discovers features such as Kmer, RevKmer and features based on correlation between di/tri nucleotides. None of these are close to finding the features we need, which are all the motifs and their positions. Other two components, Pse RAC—General accepts RNA sequence as input and Pse AAC-General takes input of protein sequences. The method proposed analyses the sequence of motifs. Pse-in One is not designed to take this as input and hence is not suited for our type of analysis.

The inherent high dimension of the data leads to the problems of difficulty in analysis and inaccuracy in the results of analysis. This is mostly due to the noise, in the form of redundant information embedded in the features. Dimensionality reduction procedures are thus an essential step in the analysis of large dimension data sets. Feature selection and feature transformation are two common methods for this step of dimensionality reduction. Selection of features is a simple and often efficient technique. Although feature selection improves the performance of the data mining algorithm, there is always a possibility of missing out some important features in the process. There are several approaches proposed in literature for feature selection which can be categorized as filter methods, wrapper methods and embedded methods. Filter methods select a subset of the features irrespective of the classification model used, whereas wrapper methods consider the model hypotheses to select a feasible subset. The embedded approach is also classifier dependent but is computationally less expensive compared to wrappers [12]. In this work, the significant features, from the view point of getting a good classification, are selected by filtering.

The sequential nature of the features imposes constraints on classification of biological sequences, hence making it a challenging task as compared to classification of feature vectors [13]. There have been a number of successful attempts in the past for finding the similarity in the coding as well as the non-coding regions of the DNA sequences. The two major tasks involved in this process are alignment and analysis. A variety of computational models exist for alignment such as Bayesian Methods [14], Scoring Matrices [15,16], Dot Matrix [17], Dynamic Programming [18] and Genetic Algorithms [19]. Nevertheless, most of these methods are based on nucleotide comparisons, which can be useful in various contexts. Motifs / transcription factor binding sites (TFBSs) are known to be important patterns within the promoter sequences. Simple alignment of nucleotides will disperse the conserved regions of motifs and hence not suited for promoter comparisons. Analyzing distribution of motifs in the promoter regions, alignment of motifs are some ways of comparing promoters [20]. Study of simple distributions, such as frequency of occurrence of each motif across the promoter or simple alignment of motif sequences as traditionally done in coding regions with nucleotides, do not utilize or keep the important information of position of motifs in the promoter sequences.

The model proposed in this work uses the sequence of motifs as well as their positional information. A promoter is reduced to a matrix called Position Specific Motif Matrix (PSMM), where rows are motifs present in the promoters and columns are positions where these motifs are present. This PSMM written as a single row (concatenation of rows of PSMM) is the feature vector of a promoter. A matrix of feature vectors of all promoters is the feature matrix of the set of promoters and the classification is performed on the feature matrix.

## Materials and Methods

This section describes the proposed methods and the data sets used to test the proposed methods.

### Overall schema of the proposed model

**Fig 1:**
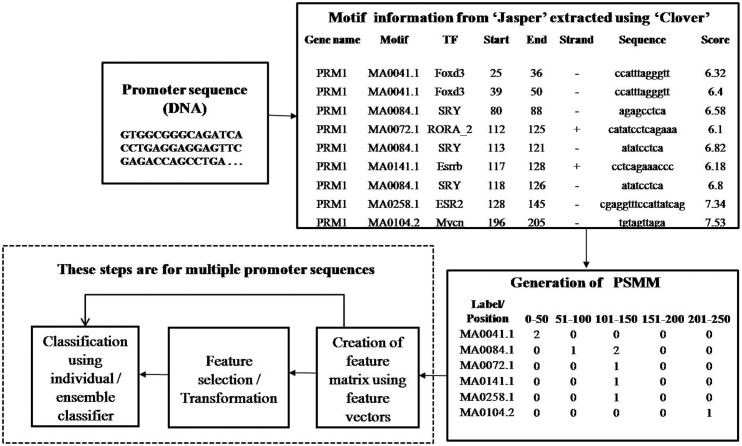
Overall schema and flow of the method.

The overall schema and flow of the method is as described in Fig 1. The construction of the PSMM for a promoter has been described earlier [7,21]. Using the PSMMs of all promoters, feature matrix is identified. Classification of promoters is performed using (i) all features (ii) features with high variances (iii) features with low P values and (iv) MRMR [22]. Also, classification is performed using transformed features such as PCA [23] and SVD [24] which are frequently used transformations in literature. We carried out experiments using these transformed features as a comparative study. In this work, we have experimented with three individual classification techniques viz., KNN, SVM and Decision Trees and an ensemble classifier named LibD3C [25].

### Classification algorithms

There have been several attempts in the recent past to efficiently classify biological data to aid biologists in different tasks and solve some specific biological problems [26,27]. The classification capability is greatly influenced by the method adopted and the choice of parameters [28,29]. Some popular classification techniques like Bayesian classification, Hidden Markov Models [HMMs], Support Vector Machine [SVM] and Decision Trees [DT] have been used in the recent past for biological sequence classification. SVM is used for successful classification and validation of cancer tissues using the micro array expression data [30]. Recursive feature elimination based SVM (RFE-SVM) is yet another successful example in classification of gene expression data [31]. Human DNA sequence prediction is performed using the Bayesian classification in [14,32]. Motifs-based HMMs have been successfully employed for classification of genes using the promoter regions [33]. KNN is a lazy learning method that classifies an unseen sample by vote of k-nearest training instance by using a distance metric, typically Euclidean distance [34]. The choice of the distance measure is critical to the performance of KNN classifiers [13]. KNN estimates the density function for every target instance sample locally and differentially instead of estimating once for the entire instance space [34].

SVM is another popular classification method which is proven to be effective for sequence classification [35,36,37]. The two significant challenges encountered while using SVM for sequence classification are, definition of kernel functions and computational efficiency of kernel matrices [13]. SVM performs well when a simple kernel is used for a small data. Use of more complex kernels may become necessary when datasets containing more samples become available [30]. Weston et al [38] propose a semi-supervised protein classification method by incorporating a cluster kernel into the SVM and they claim that the cluster kernel works better by adding unlabeled data than when using only the labeled data.

The other method used in this work for classification is the Decision tree. Decision tree is one of the most popular technique used by the machine learning community in general [39,40] and particularly has applications in computational biology and bioinformatics because of their capability in aggregating diverse types of data to make an accurate prediction [41]. Decision trees are sometimes more interpretable and can be trained more efficiently than other classifiers like SVM and Neural Networks because they combine simple questions about the data in an understandable way [41]. Also, decision trees suffer less from the curse of dimensionality [39, 40]. However, small changes in the input data can sometimes lead to large change in the constructed tree.

LibD3C [25] is an ensemble classifier, this approach is a hybrid model of ensemble pruning that is based on k-means clustering and the framework of dynamic selection and circulating in combination with a sequential search method. Ensemble classifier pruning becomes useful in some applications, where the number of independent classifiers that are needed to achieve reasonable accuracy is enormously large [42].

### Creation of feature matrix

**Fig 2:**
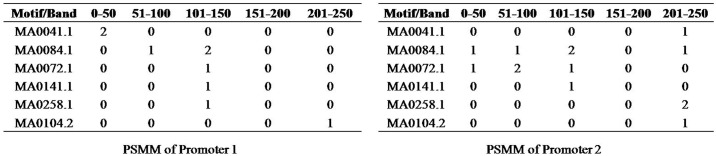
PSMMs of two promoters/samples.

**Fig 3:**
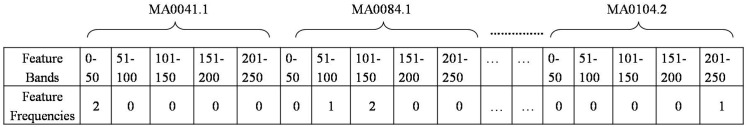
Feature vector of promoter 1.

**Fig 4:**
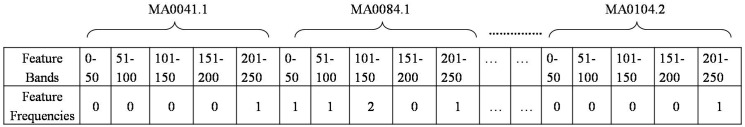
Feature vector of promoter 2.

The PSMM of a promoter/sample is a row in the feature matrix. The successive rows of PSMM are appended to get a single row in the feature matrix. The PSMM of the promoter/sample in the Fig 1 along with the PSMM of another sample is shown in Fig 2. The feature vectors of the two promoters 1 and 2 in Fig 2 are shown in Figs 3 and 4 respectively. In the proposed promoter analysis, position and frequencies of the transcription factor binding sites (TFBSs)/motifs are the features. The design of feature matrix keeps this information intact.

### Reduction of features

Applying feature selection techniques in bioinformatics has become a prerequisite for model building [12]. The major advantages of feature selection are (i) it improves the performance of the model, (ii) it provides faster and more cost effective models and (iii) it helps gain a deeper insight into the underlying processes [12]. As feature selection merely selects a subset of features, it does not change the actual representation of the features [12], hence preserves the original semantics which can be easily interpreted by a domain expert [12]. MRMR [22] is one of the most robust feature selection techniques that is useful in various applications. MRMR-MIQ features compute the significance of each feature one by one and rank the features according to their significances in the descending order [43].

Often in classification problems, features are transformed and later features are selected in the transformed space. However, there are some advantages in reduction of original features. The reduced feature set can be useful information to the biologists, since it points to key motifs and their positions that are significant in differentiating the promoter sets. In the transformed space this kind of inference is not possible. PCA and SVD are methods of this type and frequently used in literature. PCA and SVD are the basic linear transformations of the input variables [24]. PCA extracts the components by maximizing the variance of a linear combination of the original features [23]. We have experimented and compared the efficiency of the proposed feature selection methods using these popular methods. The section next gives an overview of the proposed feature selection methods. For reasons mentioned above, our selection methods do not transform features.

#### Variance based reduction

**Fig 5:**
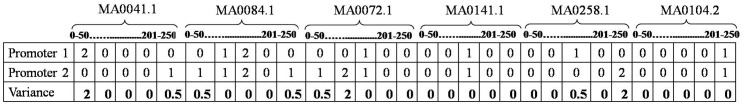
Example feature matrix of PSMMs of promoters 1 and 2.

If the number of promoters considered for analysis is just two, then the feature matrix is as shown in Fig 5. Features are selected based on variance. We find variance of every column (features) and those that are highly variant are selected. Total variance in all features is computed. Features are then added on to the selected set in the decreasing order of their variations until a specified threshold P% of variation is covered as described in Eq (2). Rest of the features are ignored. By doing so, we select not only motifs but also specific regions of the motif. A motif in a specific position may get selected and the same motif in some other positions may be ignored.

Suppose, v_1_, v_2_, v_3_, v_4_ ……. v_n_ are the variances of ‘n’ features. Then, the total variance (T_V_)of the n features is given by,

$${\text{T}}_{\text{v}}\;=\sum_{\text{j}=1}^{\text{n}}{\text{V}}_{\text{i}}$$ (1)

Let j_1_, j_2_, j_3_, j_4_ ……………. j_k_ be the features selected, where k<< n.

Then, Var j_1_ ≥ Var j_2_ ≥ Var j_3_ ≥ Var j_4_…………………≥Var j_k_

$$\sum_{\text{i}=1}^{\text{k}}\text{Var}\left({\text{j}}_{\text{i}}\right)\geq \left(\text{P}\times {\text{T}}_{\text{v}}\right)÷100$$ (2)

and

$$\sum_{\text{i}=1}^{\text{k}-1}\text{Var}\left({\text{j}}_{\text{i}}\right)<\left(\text{P}\times {\text{T}}_{\text{v}}\right)÷100$$

For example, in the feature matrix in Fig 5, the total variance is 8.5. Let P = 50%, then 50% of 8.5 is 4.25. Hence, only the features ‘MA0041.1’ in band 0–50, ‘MA0072.1’ in band 51–100 and ‘MA0258.1’ in band 201–250 are selected since the sum of variations is 6, which is just greater than 4.25, the threshold for selection in this case. Thus, ‘MA0041.1’is selected in the region 0–50 whereas the same motif is ignored in other regions.

Advantage of variance based reduction of features is, it is computationally simple and generally it works very well for moderately separated classes. If the data is known to have a lot of overlap of classes, T test based reduction will perform better than simple variance based reduction. This is because individual class means and variances are used in the process of reduction.

#### P value based reduction

Typically, we classify two or more sets of promoters using the selected features. In biological applications P values are important and often used in variety of applications. P values of features are calculated based on t distribution. Features with lower P values are better since these indicate presence of two distinct classes. A threshold on the number of features (T %) is set. Features in the increasing order of P values are added to the list until T% is selected as described in Eq (3).

Suppose, p_1_, p_2_, p_3_, p_4_ …….. p_n_ are the P values of ‘n’ features.

Let l_1_, l_2_, l_3_, l_4_ ……………. l_k_ be the features selected, where k << n.

Then, pl_1_ ≤ pl_2_ ≤ pl_3_≤ pl_4_…………………≤ pl_k_

The number of features selected ‘k’ is

k =(n×T)÷100 (3)

**Fig 6:**
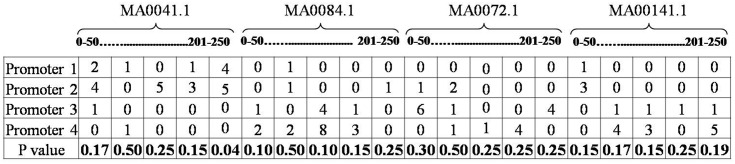
Hypothetical feature matrix of PSMMs of 4 promoters from two classes and their P values.

For example, consider a feature matrix of 4 promoters from 2 different sets as shown in Fig 6. Suppose that the first two promoters belong to set/class 1 and next two to set/class 2. T test is performed on values of set 1 and set 2 across all the columns as shown in Fig 6. Suppose, the threshold T = 50% then the number of features selected is 10 (50% of total number (20) of features) in the increasing order of their P values. Therefore, the selected features for this particular example are motif MA0041.1 in band 51–100, 151–200 and 201–250 with P values 0.17, 0.15 and 0.04. MA0084.1 in bands 0–50, 101–150 and 151–200 with p values of 0.10, 0.10 and 0.15. MA00141.1 is selected in 4 of 5 bands except band 151–200 with P values of 0.15, 0.17, 0.15 and 0.19. Rest of the bands (also motifs—in this example MA0072.1) are ignored as they do not satisfy the threshold conditions.

### Dataset description

Here we describe the origin and selection of data sets to test the proposed methods. Dataset 1 contains 6 sets (one test and 5 backgrounds) having 124 promoters in each set. Dataset 2 has 3 sets having 100 promoters in each set.

#### Dataset 1

Among 176 genes listed in the supplementary notes of experiments on transcriptional regulation of HL60 neutrophil differentiation [44], 124 genes were selected with known functional genes and extracted promoter sequences for these genes using UCSC chromosomal sequences, BioMart annotations and a PERL program. Similarly, five background sets of promoters of genes, which were known to be not differentially expressed, were also obtained. Using Clover tool [45], JASPAR [46] matrices were scanned to obtain the motif information of the promoter as shown in Fig 1.

#### Dataset 2

Ubiquitous and tissue-specific gene lists:

The ubiquitous gene list was obtained from an earlier report [47]. We also used the list of genes transcribed in the Testis, Uterus and Kidney from three recent bio curated mammalian gene expression databases MGEx-Tdb, MGEx-Udb and MGEx-Kdb respectively. The advantage of these databases is that the genes were assigned a reliability score based on a meta-analysis of multiple data sets such that the score for a gene indicates the consistency of its transcription status across experiments. Cumulative reliability scores from the 3 databases were used, to hierarchically list the ubiquitous genes. Thus, ubiquitous genes from the earlier report were short-listed if they were also present in 3 tissues considered, with high reliability scores, according to the MGEx-dbs.

Testis and kidney transcribed lists from MGEx-Tdb and MGEx-Kdb were also similarly used to derive a hierarchical list of tissue-specific genes. Testis and kidney specific genes were first obtained from the TiGER database [48] with EST enrichment value, Refseq IDs. Testis-specific genes from the TiGER database that were also transcribed according to the MGEx-Tdb were then short-listed. Similarly, the kidney-specific genes from TiGER database were also short-listed using MGEx-Kdb. But both EST enrichment scores (scaled 0–10) and the reliability score (scaled 0–10) were added and the sum used to sort the tissue-specific genes.

For the top 100 (ubiquitous/tissue-specific) genes, respective ensemble transcript ID was obtained using ensemble. Then, the promoter sequences (-2000 upstream and +500 downstream) corresponding to the selected genes were retrieved using the MGEx databases.

## Experiments and Results

The experiments were conducted with complete feature set and also with selected features. As mentioned in the earlier section, selection of features is done using two criteria namely variances and P values. In case of variance based reduction, a threshold on total variation is set for selecting features. Features with higher variance are sequentially added to the selection list until the sum of variations of features in the selected set is just greater than the threshold. With P value selection, the threshold is chosen on the percentage (T%) of features to be selected. Features are added to the selection list in the increasing order of P value until T% is included in the list.

Features selection using MRMR, PCA and SVD are also explored. For these selection methods, the available packages are used. MRMR is a selection procedure. PCA and SVD perform transformation of features and then features are selected.

Classification is performed using three classifiers (KNN, SVM, Decision Tree) and an ensemble classifier (LibD3C) for various parameter settings (such as different K for KNN, different kernels for SVM and for various learning, testing ratios). Details of the extensive experiments conducted on the two datasets are given in Table 1.

**Table 1:** Details of number of experiments. (Dataset 1 has 5 pairs of promoter sets and Dataset 2 has 3 pairs of promoter sets).

Feature reduction / Selection method	Classifier		
	KNN	SVM	Decision Tree
**Complete Features**	5 Ks x 5 L:T ratios with all features = 25	5 kernels x 5 L:T ratios with all features = 25	5 with all features
	Total = 25 x 8 pairs	Total = 25 x 8 pairs	Total = 5 x 8 pairs
**Feature variance**	5 Ks x 5 L:T ratios x 5 levels of reduction = 125	5 kernels x 5 L:T ratios x 5 levels of reduction = 125	5 L:T ratios x 5 levels of reduction = 25
	Total = 125 x 8 pairs	Total = 125 x 8 pairs	Total = 25 x 8 pairs
**P value of features**	5 Ks x 5 L:T ratios x 5 levels of reduction = 125	5 kernels x 5 L:T ratios x 5 levels of reduction = 125	5 L:T ratios x 5 levels of reduction = 25
	Total = 125 x 8 pairs	Total = 125 x 8 pairs	Total = 25 x 8 pairs
**MRMR**	5 Ks x 5 L:T ratios x 5 levels of reduction = 125	5 kernels x 5 L:T ratios x 5 levels of reduction = 125	5 L:T ratios x 5 levels of reduction = 25
	Total = 125 x 8 pairs	Total = 125 x 8 pairs	Total = 25 x 8 pairs
**PCA**	5 Ks x 5 L:T ratios x 5 levels of reduction = 125	5 kernels x 5 L:T ratios x 5 levels of reduction = 125	5 L:T ratios x 5 levels of reduction = 25
	Total = 125 x 8 pairs	Total = 125 x 8 pairs	Total = 25 x 8 pairs
**SVD**	5 Ks x 5 L:T ratios x 5 levels of reduction = 125	5 kernels x 5 L:T ratios x 5 levels of reduction = 125	5 L:T ratios x 5 levels of reduction = 25
	Total = 125 x 8 pairs	Total = 125 x 8 pairs	Total = 25 x 8 pairs
**Total number of experiments**	**5200**	**5200**	**1040**

We also performed experiments using one of the popular ensemble classifier LibD3C for the best performing feature sets (P value based reduced features and MRMR reduced features) on individual classifiers.

### Results of experiments on dataset 1

The classification results using different classification techniques (KNN, SVM and Decision Trees) for different learning testing ratios on this dataset are summarized in the Tables 2 to 7. The experiment was repeated 25 times for randomly selected training and testing samples and the average accuracy of these 25 experiments is quoted. We observe in Table 2 that the performance of KNN is consistent irrespective of the K values (1 to 5). SVM classification technique was performed with different kernels (Linear, Quadratic, Polynomial, RBF-Radial Basis Function and MLP-Multilayer Perceptron). It can be seen in Table 3 that Polynomial and MLP kernels do not give satisfactory classification accuracy when compared to the other three kernels when the dimension of features is very high. However, we could observe (Table 3) that their performance significantly improves when the input of the dimension of features is very low (5% of total variation).

**Table 2:** KNN Classification Results for Test v/s Background1 (Variance Reduced).

K	K = 1				K = 2				K = 3				K = 4				K = 5			
P(%) Ratios	100%	50%	10%	5%	100%	50%	10%	5%	100%	50%	10%	5%	100%	50%	10%	5%	100%	50%	10%	5%
**50–50**	0.76	0.8	0.87	0.94	0.77	0.78	0.87	0.94	0.75	0.8	0.86	0.94	0.76	0.79	0.87	0.94	0.76	0.79	0.86	0.94
**60–40**	0.81	0.84	0.9	0.95	0.81	0.83	0.88	0.95	0.81	0.85	0.89	0.95	0.82	0.84	0.89	0.96	0.78	0.84	0.9	0.96
**70–30**	0.85	0.86	0.92	0.97	0.85	0.88	0.92	0.97	0.87	0.88	0.92	0.97	0.85	0.87	0.92	0.97	0.86	0.88	0.91	0.97
**80–20**	0.92	0.91	0.95	0.98	0.9	0.92	0.96	0.98	0.92	0.91	0.94	0.98	0.9	0.91	0.95	0.98	0.92	0.91	0.94	0.98
**90–10**	0.96	0.96	0.98	0.99	0.95	0.96	0.97	0.99	0.96	0.95	0.97	0.99	0.95	0.97	0.97	0.99	0.95	0.97	0.98	1

**Table 3:** SVM Classification Results for five different kernels for Test v/s Background1 (Variance Reduced).

Kernel	Linear				Quadratic				Polynomial				RBF				MLP			
P(%) Ratios	100%	50%	10%	5%	100%	50%	10%	5%	100%	50%	10%	5%	100%	50%	10%	5%	100%	50%	10%	5%
**50–50**	0.75	0.81	0.99	1	0.73	0.74	0.79	0.88	0.48	0.51	0.79	0.95	0.74	0.74	0.74	0.84	0.49	0.53	0.68	0.81
**60–40**	0.8	0.86	1	1	0.8	0.8	0.84	0.91	0.8	0.51	0.84	0.97	0.81	0.78	0.79	0.89	0.81	0.53	0.68	0.82
**70–30**	0.86	0.9	1	1	0.83	0.85	0.88	0.94	0.52	0.51	0.9	0.98	0.84	0.84	0.85	0.92	0.43	0.53	0.68	0.84
**80–20**	0.91	0.93	1	1	0.88	0.89	0.91	0.97	0.48	0.5	0.93	0.98	0.89	0.89	0.89	0.94	0.44	0.5	0.69	0.84
**90–10**	0.95	0.95	1	1	0.93	0.95	0.96	0.98	0.52	0.5	0.95	0.99	0.93	0.96	0.94	0.99	0.39	0.52	0.68	0.83

**Table 4:** Decision Tree Classification Results for Test v/s Background1 (Variance Reduced).

P(%) Ratios	100%	50%	40%	30%	20%	10%	5%
**50–50**	0.742258	1	1	1	1	1	1
**60–40**	0.778586	1	1	1	1	1	1
**70–30**	0.811892	1	1	1	1	1	1
**80–20**	0.869388	1	1	1	1	1	1
**90–10**	0.945	1	1	1	1	1	0.998333

**Table 5:** KNN Classification Results for K = 1 for test v/s all five backgrounds (Variance Reduced).

Dataset	Test_Bg1				Test_Bg2				Test_Bg3				Test_Bg4				Test_Bg5			
P(%) Ratios	100%	50%	10%	5%	100%	50%	10%	5%	100%	50%	10%	5%	100%	50%	10%	5%	100%	50%	10%	5%
**50–50**	0.76	0.8	0.87	0.94	0.76	0.8	0.91	0.97	0.75	0.78	0.51	0.5	0.74	0.78	0.89	0.96	0.77	0.77	0.89	0.96
**60–40**	0.81	0.84	0.9	0.95	0.78	0.84	0.94	0.97	0.81	0.83	0.51	0.5	0.8	0.82	0.91	0.97	0.82	0.81	0.92	0.97
**70–30**	0.85	0.86	0.92	0.97	0.85	0.88	0.94	0.98	0.86	0.88	0.51	0.51	0.85	0.88	0.94	0.98	0.87	0.86	0.93	0.97
**80–20**	0.92	0.91	0.95	0.98	0.91	0.91	0.96	0.99	0.91	0.92	0.5	0.53	0.89	0.92	0.95	0.98	0.91	0.9	0.95	0.98
**90–10**	0.96	0.96	0.98	0.99	0.95	0.97	0.98	1	0.96	0.95	0.51	0.51	0.95	0.96	0.98	1	0.96	0.94	0.98	1

**Table 6:** SVM Classification Results for Linear Kernel for test v/s all five backgrounds (Variance Reduced).

Dataset	Test_Bg1				Test_Bg2				Test_Bg3				Test_Bg4				Test_Bg5			
P(%) Ratios	100%	50%	10%	5%	100%	50%	10%	5%	100%	50%	10%	5%	100%	50%	10%	5%	100%	50%	10%	5%
**50–50**	0.75	0.81	0.99	1	0.74	0.89	1	1	0.75	0.82	0.5	0.5	0.73	0.81	1	1	0.75	0.79	0.95	0.98
**60–40**	0.8	0.86	1	1	0.8	0.92	1	1	0.79	0.87	0.49	0.51	0.78	0.84	1	1	0.79	0.83	0.97	0.96
**70–30**	0.86	0.9	1	1	0.85	0.96	1	1	0.86	0.92	0.51	0.5	0.83	0.89	1	1	0.85	0.89	0.99	0.95
**80–20**	0.91	0.93	1	1	0.9	0.99	1	1	0.9	0.95	0.53	0.5	0.89	0.92	1	1	0.92	0.91	0.98	0.96
**90–10**	0.95	0.95	1	1	0.94	1	1	1	0.95	0.98	0.52	0.5	0.96	0.96	1	1	0.95	0.96	0.99	0.95

**Table 7:** Decision Tree Classification Results for test v/s all five backgrounds (Variance Reduced).

Dataset	Test_Bg1				Test_Bg2				Test_Bg3				Test_Bg4				Test_Bg5			
P(%) Ratios	100%	50%	10%	5%	100%	50%	10%	5%	100%	50%	10%	5%	100%	50%	10%	5%	100%	50%	10%	5%
**50–50**	0.74	1	1	1	0.75	1	1	1	0.73	1	0.51	0.51	0.73	1	1	1	0.73	0.99	0.99	0.99
**60–40**	0.78	1	1	1	0.78	1	1	1	0.78	1	0.51	0.52	0.78	1	1	1	0.78	0.99	1	1
**70–30**	0.81	1	1	1	0.83	1	1	1	0.83	1	0.53	0.5	0.83	1	1	1	0.83	1	0.99	0.99
**80–20**	0.87	1	1	1	0.88	1	1	1	0.86	1	0.51	0.51	0.88	1	1	1	0.87	1	1	0.99
**90–10**	0.95	1	1	1	0.9	1	1	1	0.93	1	0.51	0.51	0.91	1	1	1	0.93	1	0.99	0.99

We can see in Table 8 that PCA/SVD features perform poor when compared to original features. With PCA/SVD features, only for very large size learning set, the classification accuracy of these features is comparable to that of the original features. MRMR yields very good results for all L: T ratios and all reduction levels. However, almost same accuracy for most cases is obtained even with simple selection procedures based on variance and P values.

**Table 8:** Selected classification results of KNN, SVM and Decision Trees for the 3 sets of promoters of dataset 1for learning testing ratio of 60:40 for different feature selection/transformation methods (File 1: Test v/s Background1, File 2: Test v/s Background2, File 3: Test v/s Background3, File 4: Test v/s Background4, File 5: Test v/s Background5).

Dataset and Classifier	Complete Features	Variance Reduction		P value Reduction		MRMR		PCA		SVD	
**File 1**	**100%**	**50%**	**10%**	**50%**	**10%**	**50%**	**10%**	**50%**	**10%**	**50%**	**10%**
**KNN**	0.81	0.84	0.9	0.66	0.63	0.87	0.98	0.81	0.83	0.8	0.8
**SVM**	0.8	0.86	1	1.00	0.99	0.99	1	0.82	0.81	0.81	0.81
**DT**	0.78	1	1	1.00	0.82	1	1	0.84	0.77	0.8	0.76
**File 2**	**100%**	**50%**	**10%**	**50%**	**10%**	**50%**	**10%**	**50%**	**10%**	**50%**	**10%**
**KNN**	0.78	0.84	0.94	0.65	0.59	0.91	0.99	0.79	0.8	0.8	0.82
**SVM**	0.8	0.92	1	0.99	0.98	0.99	1	0.77	0.77	0.79	0.8
**DT**	0.78	1	1	1.00	0.83	1	1	0.83	0.78	0.78	0.79
**File 3**	**100%**	**50%**	**10%**	**50%**	**10%**	**50%**	**10%**	**50%**	**10%**	**50%**	**10%**
**KNN**	0.81	0.83	0.51	0.69	0.67	0.95	1	0.82	0.81	0.81	0.8
**SVM**	0.79	0.87	0.49	0.99	0.98	0.99	1	0.81	0.51	0.82	0.72
**DT**	0.78	1	0.51	1.00	0.85	1	1	0.83	0.71	0.8	0.76
**File 4**	**100%**	**50%**	**10%**	**50%**	**10%**	**50%**	**10%**	**50%**	**10%**	**50%**	**10%**
**KNN**	0.8	0.82	0.91	0.59	0.61	0.87	0.96	0.79	0.81	0.8	0.79
**SVM**	0.78	0.84	1	0.99	0.99	0.99	1	0.82	0.78	0.83	0.8
**DT**	0.78	1	1	1.00	0.82	1	1	0.85	0.79	0.8	0.78
**File 5**	**100%**	**50%**	**10%**	**50%**	**10%**	**50%**	**10%**	**50%**	**10%**	**50%**	**10%**
**KNN**	0.82	0.81	0.92	0.62	0.62	0.91	0.96	0.83	0.8	0.81	0.8
**SVM**	0.79	0.83	0.97	1.00	0.99	1	1	0.81	0.77	0.82	0.81
**DT**	0.78	0.99	1	1.00	0.83	1	1	0.84	0.78	0.8	0.76

In Table 9 the classification performance of ensemble classifier LibD3C using the best features MRMR and P value is presented. When compared to all three individual classifiers, the overall classification accuracies are poor for all the experiments conducted. Ensemble classifiers generally perform better than individual classifiers, but could fail occasionally. This could be because of the k—means clustering algorithm used in LibD3C can be instable and because some classifiers with useful information are excluded from the ensemble without multilayer optimization [25]. Also the time taken by LibD3C is generally more than other individual classifiers.

**Table 9:** LibD3C classification accuracies for MRMR and P value reduced features on dataset 1.

File1/percentage of features	P value Reduced	MRMR Reduced
**100%**	49.60	49.60
**50%**	65.73	77.02
**40%**	74.19	80.65
**30%**	74.60	77.82
**20%**	76.61	82.66
**10%**	80.65	81.85

**Fig 7:**
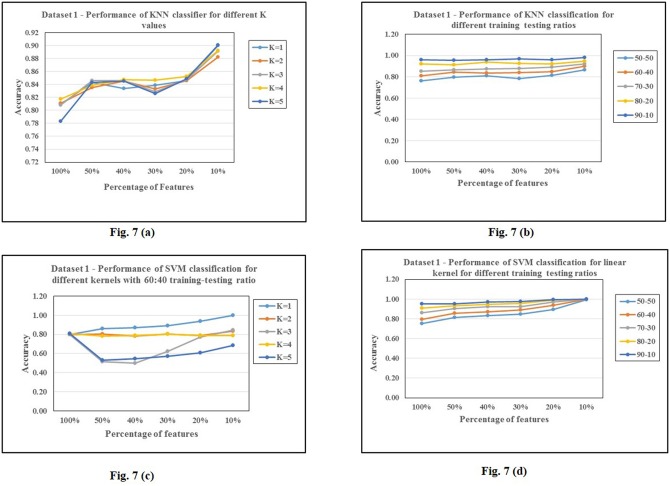
Analysis of classification accuracies for various parameters on dataset 1.7 (a), 7(b): KNN, 7 (c), 7(d): SVM.

**Fig 8:**
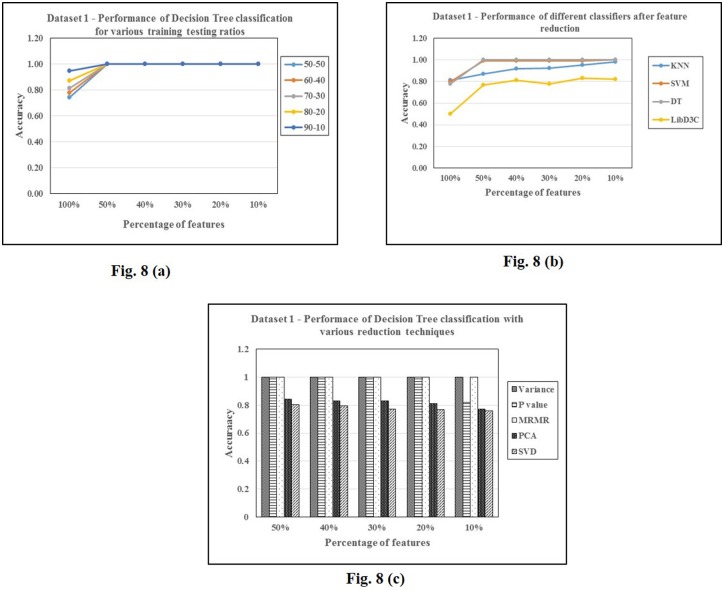
Analysis of classification accuracies on dataset 1.8 (a): Decision Trees.8 (b): different classifiers 8(c): different feature selections/transformations.

For background 1, the summary of results after selecting features based on their variance is given in Tables 2, 3 and 4. We observe that decision trees outperform KNN and SVM particularly when the features are reduced. Tables 5, 6 and 7 show the performance of the classifiers for test v/s different backgrounds. We can notice that all the backgrounds show similar performance on all the three classification techniques except background 3. This background shows a drastic fall in number of features as well as accuracy for a threshold<30% of total variance, irrespective of the classifier used. This difference in the background 3 is perhaps due to the presence of a few highly variant features which can be observed from Table 10. Figs 7 and 8 show some plots of classification accuracies obtained for various parameters of classifiers and for different classifiers and feature selections/transformations.

**Table 10:** Feature reduction (Variance) pattern for test v/s 5 backgrounds of dataset 1.

Threshold	Test_Bg1	Test_Bg2	Test_Bg3	Test_Bg4	Test_Bg5
100%	5496	5536	5618	5495	5495
50%	1109	1037	621	1069	1049
40%	790	728	319	755	739
30%	515	468	98	490	476
20%	286	251	14	269	258
10%	102	81	2	90	84
5%	33	21	1	26	23

### Results of experiments on dataset 2

The experimental setup is same as that of Dataset 1 as described in the earlier section. Selected results are shown in Table 11. Highlights of the results obtained in this extensive experimentation are given in S3 File. The detailed results on top 100promoter sets as well as the complete results of dataset 1 are given in S1 and S2 Files.

**Table 11:** Selected classification results of KNN, SVM and Decision Trees for the 3 sets of promoters of dataset 2for learning testing ratio of 60:40 for different feature selection/transformation methods (File 1: Kidney v/s Ubiquitous, File 2: Testis v/s Ubiquitous, File 3: Kidney v/s Testis).

Dataset and Classifier	Complete Features	Variance Reduction		P value Reduction		MRMR		PCA		SVD	
**File 1**	**100%**	**50%**	**10%**	**50%**	**10%**	**50%**	**10%**	**50%**	**10%**	**50%**	**10%**
**KNN**	0.8	0.51	0.58	0.49	0.57	0.81	0.85	0.8	0.75	0.8	0.82
**SVM**	0.84	0.88	0.94	1	1	0.99	0.99	0.82	0.82	0.84	0.82
**DT**	0.79	1	1	1	1	1	1	0.89	0.86	0.85	0.8
**File 2**	**100%**	**50%**	**10%**	**50%**	**10%**	**50%**	**10%**	**50%**	**10%**	**50%**	**10%**
**KNN**	0.82	0.68	0.56	0.7	0.53	0.82	0.88	0.82	0.79	0.8	0.82
**SVM**	0.84	0.87	0.96	1	1	0.99	0.99	0.81	0.83	0.85	0.83
**DT**	0.78	1	1	1	1	1	1	0.88	0.85	0.84	0.79
**File 3**	**100%**	**50%**	**10%**	**50%**	**10%**	**50%**	**10%**	**50%**	**10%**	**50%**	**10%**
**KNN**	0.82	0.51	0.58	0.55	0.56	0.83	0.91	0.8	0.79	0.82	0.8
**SVM**	0.8	0.83	0.93	1	1	1	0.99	0.81	0.81	0.83	0.8
**DT**	0.78	1	1	1	1	1	1	0.89	0.86	0.78	0.86

**Fig 9:**
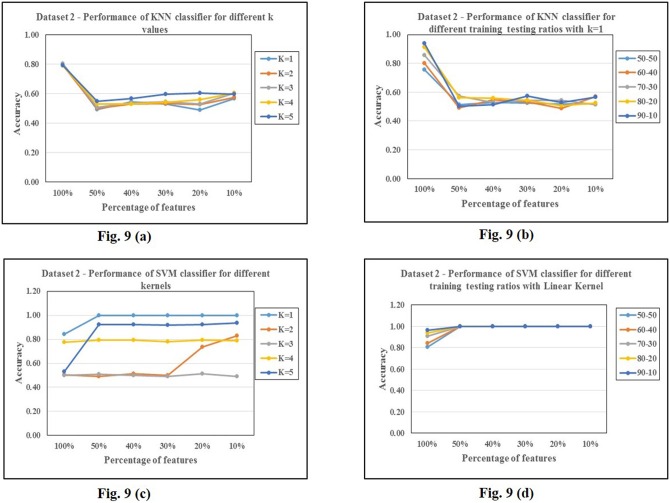
Analysis of classification accuracies for various parameters on dataset 2. 9(a), 9(b): KNN, 9(c), 9(d): SVM.

**Fig 10:**
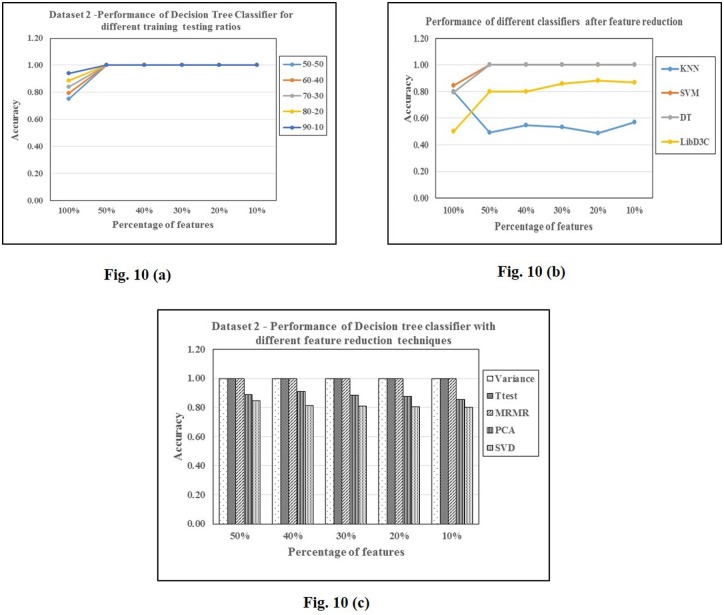
Analysis of classification accuracies on dataset 2. 10 (a): Decision Trees. 10 (b): different classifiers 10 (c): different feature selections/transformations.

The results with P value reduced features are consistently better in all cases, compared to variance reduced features as the number of features is significantly large in P value reduced features compared to variance reduced. MRMR performs best when compared to other feature selections/transformations even on dataset 2. The results of the ensemble classifier LibD3C on dataset 2 is detailed in Table 12. The pattern of feature reduction for different files of dataset 2 is presented in Table 13. Figs 9 and 10 are graphs showing classification accuracies of a classifier and of different classifiers.

**Table 12:** LibD3C classification accuracies for MRMR and P value reduced features on dataset 2.

File1/percentage of features	P value	MRMR
	Reduced	Reduced
**100%**	50	50
**50%**	80	78
**40%**	80	78
**30%**	86.5	75.5
**20%**	88	77.5
**10%**	87.5	86.5

**Table 13:** Feature reduction (Variance) pattern for 3 files of dataset 2.

Threshold	File1	File 2	File 3
100%	23800	23800	23800
50%	10695	10431	10791
40%	6192	6019	6273
30%	3120	3031	3180
20%	1236	1212	1270
10%	305	313	314
5%	56	65	57

### Implementation details

The experiments were carried out on an Intel Core i5—4460 @ 3.20 GHz machine with 4GB RAM. The code is implemented in MATLAB. It is empirically shown that, reduction in features improves classification accuracy. From Table 14, it is also evident that reduction in features results in drastic reduction in the execution time. This becomes important when we are dealing with large data sets. The time for KNN is the total time taken for K values from 1 to 5 and in the case of SVM it is the total time taken for execution of all 5 kernels. The pattern in reduction of time is same for both KNN and Decision Trees, but in the case of SVM the reduction in time is not uniform, this is due to the convergence issue that exists with some of the kernels used.

**Table 14:** Execution time (in seconds) for different classifiers for features of different thresholds for the experiment test v/s background 1 (Dataset 1).

		Classifier Used		
Threshold(P)	No. of features	KNN (k = 1 to 5)	SVM (all 5 kernels)	Decision Trees
100%	5495	248.17	38.18	947.08
50%	1109	37.71	22.1	12.8
40%	790	21.61	22	9.8
30%	515	11.35	21	6.6
20%	286	8.17	25	4.22
10%	102	5.85	51	2.28
5%	33	4.70	15.28	1.59

The CPU time analysis for the two datasets is shown in Tables 15 and 16. It is clear MRMR takes most time for all feature set sizes of dataset 1. On the other hand, PCA consumes more time than MRMR when the feature set size becomes smaller. However, Variance and P value based reductions are most time efficient.

**Table 15:** CPU time taken by various reduction methods in seconds for Test v/s Background1 file of dataset 1.

Percentage of features	Variance	P value	MRMR	PCA	SVD
5%	0.0429	2.5092	13.1602	1.4172	1.1232
10%	0.0476	2.5020	40.1155	1.4293	1.1113
20%	0.0645	2.9271	113.0153	1.4660	1.1416
30%	0.1396	3.5771	201.8241	1.5421	1.1381
40%	0.2471	4.5635	317.6056	1.6630	1.1146
50%	0.5328	5.8676	480.5974	1.6032	1.1059

**Table 16:** CPU time taken by various reduction methods in seconds for Kidney v/s Ubiquitous file of dataset 2.

Percentage of features	Variance	P value	MRMR	PCA	SVD
5%	0.1113	8.1954	37.7775	419.833	14.5083
10%	0.12215	9.6506	171.9365	645.02	14.6427
20%	0.3930	16.5799	726.653	854.85	14.8891
30%	1.3819	28.4595	1693.048	1327.57	14.5713
40%	3.5866	44.9828	3300.43	682.47	14.2100
50%	8.0970	66.2783	5671.32	747.10	14.4678

## Conclusion

The results obtained indicate that the variance based and P value based feature selection methods can be effectively used for classifying promoter sequences. Also, we have successfully demonstrated the effect of dimensionality reduction on some of the popular classification techniques used on biological sequences for our experiments on selected promoter sequences. KNN and SVM (particularly with Linear, Quadratic and RBF kernels) perform well even when the dimensionality is very high. Discriminative ability of SVM could be highly improved with good feature selection on Polynomial and MLP kernels. Decision trees seem to be one of the best classifier that achieves good accuracy even when the data dimension is high and the accuracy marginally improves when the dimensionality decreases [27]. We observe a significant improvement in results when compared to some recent methods [6]. This is because we retain the PSMM details in the process of differentiating the promoter sets in this work. Whereas, in [6] the summary of the PSMM is used for promoter set differentiation. The proposed methods out perform some of the popular feature transformation methods such as PCA and SVD. Also, the methods proposed are as accurate as MRMR (feature selection method) but much faster than MRMR. However, we need to further explore the efficiency of this technique for different promoter datasets. Sometimes, even after feature selection using sophisticated techniques, the dimensionality of the chosen features may still be very high [13]. Hence, we can attempt to reduce the feature set by combining different feature selection techniques using ensemble feature selection approaches based on the fact that there cannot be a single universally optimal feature selection technique [49]. Also, there is a possible existence of more than one subset of features that discriminates the data equally well [50]. A combination of different classification and feature selection techniques can both lead to different results [27].

In general, using minimal features for fast classification may help to distinguish functionally different sets of promoters. Such efforts would help scientists understand the molecular mechanisms of gene expression control, which in turn would aid research in many important biological topics.

## Supporting Information

S1 File Complete results of classification on Dataset1. (XLSX)

S2 File Complete results of classification on Dataset2. (XLSX)

S3 File Summary of the experimental analysis on Dataset1 and Dataset2. (DOCX)
