# Supplementary material for: Effective Feature Selection for Classification of Promoter Sequences
Source: PLoS One. 2016 Dec 15;11(12):e0167165. doi: 10.1371/journal.pone.0167165 (PMC5158321; doi:10.1371/journal.pone.0167165)
Supplement: S3 File — (DOCX) [file pone.0167165.s003.docx]

**Experimental Analysis Summary File**

**Summary of experimental results on data set 1**

The overall observations of feature selection and classifier performance are discussed here. Most of the observations pointed out are evident from figures 7 and 8.

**KNN performance**

*Feature reduction using feature variances:*

With original features, for a reduction level and for an L: T ratio, classification accuracies are more or less same in backgrounds 1,2,4,5. Size of K has no influence on the accuracy obtained. The general trend is that accuracy increases with increase in reduction as well as increase in learning data size. However, for background 3 the improvement is steep for reduction in number of features from 100% to 30%. Further reduction shows steep fall in accuracies. This is true of all K s and all L: T ratios. This could be because of loss of some vital features in the process of reduction.

*PCA / SVD:*

Contrary to original features, reduction does not mean improvement in classification accuracy. Whereas, classification accuracy improves with increase in L: T ratio. In this aspect it is similar to original features. Also, the classification results on background 3 are similar to other backgrounds. This is another aspect where the original and transformed features differ. As far as magnitude of classification accuracies, the original and transformed features are similar (except background 3). In other words there is no significant gain for the extra work of transformation. In fact we lost something in the process of transformation, namely the information of important motifs and their positions which are capable of differentiating the classes.

*Reduction using P values:*

Results obtained are very poor when compared to variance reduced, MRMR, PCA and SVD features.

*MRMR feature reduction:*

This is capable of discriminating classes better even for small sized learning set, when compared to original, PCA, SVD features. Also, everywhere (for all parameter choices) there is some improvement when compared to previous methods.

**SVM performance**

*Feature reduction using feature variances:*

With original features, linear kernels perform slightly better than KNN particularly with low L: T ratios and lower reduction (more features) with backgrounds 1, 2, 4, 5. The general trend of increase in classification accuracy with increase in reduction is retained in this classifier too. With background 3, there is a drop in accuracy when reduction increases. Except polynomial and MLP kernels, the other kernels are comparable in performance to KNN. Reduction generally affects the classification accuracy.

*PCA / SVD:*

With PCA features, the results are more less the same as with original features. Again background 3 and polynomial, MLP kernels behave differently from cases. Classification accuracies are significantly low for more features, as compared to original feature set. With SVD features, the classification accuracies are more or less the same as in the case of KNN and better than SVM with original features, particularly over backgrounds. SVD with MLP kernel performs poor. Others are as good as KNN. The conclusion is there is no significant advantage for the extra work of transformation.

*Reduction using P values:*

P value reduction works well for linear and MLP kernels, but not good with other kernels. RBF kernel performs better for higher L and less number of features.

*MRMR feature reduction:*

With this selection, the linear and quadratic kernels out perform all other classifiers. Background 3 is not a serious problem with this feature selection. The results of MLP kernel are better when compared to other feature selection methods.

**Decision tree performance**

*Feature reduction using feature variances:*

With original feature set, the results of this classifier are significantly good when compared to KNN and SVM. Complete feature set is just as good as other two classifiers.

*PCA / SVD:*

PCA / SVD features perform poor when compared to original features. Only for very large sized learning set, we get somewhat close to the accuracy when working with original features.

*Reduction using P values:*

All levels of reduction are better when compared to full set of features. This classifier yields best classification results for small sized L and high level of reduction. However, the performance of decision trees deteriorates when using 10% or less P value features.

*MRMR feature reduction:*

MRMR yields very good results for all L: T ratios and all reduction levels. However the almost the same accuracy for most cases are available even with simple selection procedures based on variance and P values.

**LibD3C (ensemble classifier) performance**

When compared to all three classifiers, the overall classification accuracies are poor for the all the experiments conducted. Ensemble classifiers generally perform better than individual classifiers, but could fail occasionally. Also the time taken for classification is much more than other individual classifiers.

**Summary of experimental results on data set 2**

**KNN performance**

*Feature reduction using feature variances:*

With original all features, the performance improves with increase in reduction level and with increase in L: T ratio. Whereas, with reduced features, the performance is poor. This data set has lot of features (thanks to frequent updates in tool JASPAR). Perhaps the reason for drop in performance could be neglecting sizable number of features. No amount of learning seems to be enough when features are dropped.

*PCA / SVD:*

PCA / SVD features, are better with this classifier. The general trend is raise in accuracy with drop in number of features and increase in learning set size. The peak accuracy is both cases are as high as 96%. There is tremendous improvement over using original features (in case of reduction) where the same figure is just about 60%. However, with all original features the results are similar to that of PCA / SVD. So there is no gain in transformation.

*Reduction using P values:*

Results obtained are same as that of features reduced using variances.

*MRMR feature reduction:*

Results obtained out of MRMR features is as good as using PCA /SVD features, which same as using all original features.

**SVM performance**

*Feature reduction using feature variances:*

With all features, linear kernel results are best. As expected, accuracy increases with size of L. Polynomial and quadratic kernels yield poor results. When features are reduced again, the linear kernel is best out of all kernels. MLP, polynomial, quadratic kernels are not good. For large L and inclusion of very less number of features are good in case of RBF kernel.

*PCA / SVD:*

With linear kernels PCA features just as good as original and reduced set of features. P value selection performs best for this kernel. However, PCA / SVD features all but MLP kernel performs better. When linear kernel is the choice, transformation does not have any added advantage.

*Reduction using P values:*

P value reduction works well for linear and MLP kernels, but not good with other kernels. RBF kernel performs better for higher L and less number of features.

*MRMR feature reduction:*

Performance of this selection method is slightly better than reduced (based on variances) feature set but same as P value based reduction as far as linear kernel is considered. Overall MRMR works well with almost all kernels except MLP.

**Decision tree performance**

*Feature reduction using feature variances and P value reduction:*

All levels of reduction are better when compared to full set of features. This classifier yields best classification results for small sized L and high level of reduction.

*PCA / SVD:*

These features are not good as compared to original features, since the accuracy is poor for small L and yields moderately good accuracy only for large size feature set.

*MRMR feature reduction:*

MRMR feature selection is just as good as original features. For the highly complex procedure there is no advantage.
